# Recent Advances in Mechanistic Understanding of Metal-Free Carbon Thermocatalysis and Electrocatalysis with Model Molecules

**DOI:** 10.1007/s40820-023-01262-8

**Published:** 2024-02-20

**Authors:** Wei Guo, Linhui Yu, Ling Tang, Yan Wan, Yangming Lin

**Affiliations:** 1 CAS Key Laboratory of Design and Assembly of Functional Nanostructures, Fujian Institute of Research on the Structure of Matter, Chinese Academy of Sciences, Fuzhou, 350002 People’s Republic of China; 2 Xiamen Key Laboratory of Rare Earth Photoelectric Functional Materials, Xiamen Institute of Rare Earth Materials, Haixi Institute, Chinese Academy of Sciences, Xiamen, 361021 People’s Republic of China; 3 State Key Laboratory of Structural Chemistry, Fujian Institute of Research on the Structure of Matter, Chinese Academy of Sciences, Fuzhou, 350002 People’s Republic of China; 4 Institute of Urban Environment, Chinese Academy of Sciences, Xiamen, 361021 People’s Republic of China

**Keywords:** Metal-free carbon catalysts, Model catalyst, Electrocatalysis, Active site, Reaction mechanisms

## Abstract

Metal-free carbon, as the most representative heterogeneous metal-free catalysts, have received considerable interests in electro- and thermo-catalytic reactions due to their impressive performance and sustainability. Over the past decade, well-designed carbon catalysts with tunable structures and heteroatom groups coupled with various characterization techniques have proposed numerous reaction mechanisms. However, active sites, key intermediate species, precise structure–activity relationships and dynamic evolution processes of carbon catalysts are still rife with controversies due to the monotony and limitation of used experimental methods. In this Review, we summarize the extensive efforts on model catalysts since the 2000s, particularly in the past decade, to overcome the influences of material and structure limitations in metal-free carbon catalysis. Using both nanomolecule model and bulk model, the real contribution of each alien species, defect and edge configuration to a series of fundamentally important reactions, such as thermocatalytic reactions, electrocatalytic reactions, were systematically studied. Combined with in situ techniques, isotope labeling and size control, the detailed reaction mechanisms, the precise 2D structure–activity relationships and the rate-determining steps were revealed at a molecular level. Furthermore, the outlook of model carbon catalysis has also been proposed in this work.

## Highlights

- Mechanistic understandings of metal-free carbon thermocatalysis and electrocatalysis from the viewpoint of model method are summarized.
- Active sites and reaction mechanisms are discussed with a focus on in-situ techniques and 2D structure–activity relationships.
- The real contribution of each alien species, defect and edge configuration to catalytic reactions are systematically highlighted at a molecular level.

## Graphical abstract

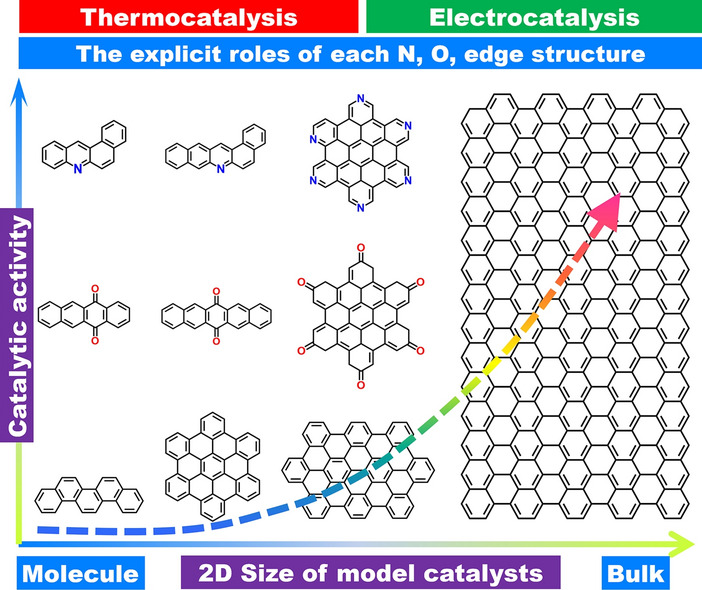

## Introduction

“Metal-free” carbon catalysts has attracted a great deal of interest for various reactions over the past two decades, including thermocatalytic hydrogen-involving and oxygen-involving reactions, electrocatalytic overall oxygen and hydrogen reactions, electrocatalytic reduction of nitrogen reactions, photocatalytic degradation reaction, to mention a few [1–17]. Usually, the established catalytic processes in chemical industries mostly use metals, in many cases, precious metals or metal oxides as “classical” catalysts [18–20]. Compared with “classical” heterogeneous catalysts, carbon catalysts are sustainable, relatively low-cost, and can avoid contamination of products due to heavy metal residues. An essential feature in heterogeneous “metal-free” carbon is the flexibility of doping with heteroatoms. O and N are the most common doping heteroatoms and can be flexibly incorporated into carbon networks by the replacement of C atoms or by saturating dangling bonds [21–34]. These heteroatoms are electronegative with respect to carbon, inducing consequently a rupture of the charge neutrality via the formation of different reactive groups (aldehydic or acid groups, phenolic, chetonic, epoxy or quinone groups, for oxygen doping and quaternary N, pyridine, amine, pyrrole for nitrogen doping, just as an example). These groups bring into redox catalytic functionalities, although often, the different stability of these functional groups (by annealing or during the catalytic reaction) is not taken into proper account. In addition, the introduction of these heteroatoms causes (i) local distortion in the structure of *sp*^2^-hybridized carbons and (ii) changes in the electron density at carbon atoms. All these aspects offer a large range of possibilities for an advanced design of catalytic sites and thus are relevant for the catalytic reactivity. Other types of heteroatoms, such as B, can accept electrons from carbon because of its electron deficiency. As a result, a shift in the Fermi level of the conducting band is induced [35, 36].

In recent years, much work has been done to investigate the effect of doping with main group non-metallic elements on the performance of carbon-based catalysts. Typically, the introduction of nucleophilic C=O group to carbon nanotubes by strong acid treatment alters the electronic localization of charges at the defect sites and thus is capable of abstracting the H atoms of alkane, improving the selectivity toward alkene in thermocatalytic dehydrogenation reaction [37–39]. This mechanistic understanding of carbon materials is important, which is a source of inspiration for the development of various efficient metal-free catalysts for selective thermocatalytic reactions. Later, the findings that doping-induced charge transfer from C atoms to the adjacent N atoms caused the change in the chemisorption mode of O_2_ to facilitate the electrocatalytic oxygen reduction reaction (ORR) on the N-doped carbon materials were reported [40]. This fundamental understanding of ORR mechanism in N-doped carbon materials gives us more possibilities to chase the catalytic application development for electrocatalytic ORR and oxygen evolution reaction (OER), and so on, via doping, and/or structural modification [41–48].

Although some mechanistic understandings for metal-free carbon catalysis have been proposed on the basis of density functional theory, advanced techniques, and plausible structure regulation, the underlying nature is still unclear due to inhomogeneities and complexities of heteroatom species associated with the morphology and surface structure of catalysts. This hampers the practical application of metal-free catalysis in chemical industries. Therefore, finding a promising strategy that is capable of determining the genuine active sites, monitoring the key intermediate products, and revealing the possible elementary reaction pathways is significant to optimize the design and development of new carbon-based catalysts. Considering that the doped carbon catalysts synthesized by traditional thermal annealing of hetero element precursors and carbon materials cannot precisely control the bonding mode of the hetero atoms, the product compositions are complicated, which hinders the in-depth study of their catalytic mechanisms. Therefore, a model catalyst with a precise molecular structure is needed as a research object. Bottom-up synthesis of large polycyclic aromatic hydrocarbons (PAH) is considered an important method for constructing model catalysts with precise structures. After the considerable investigation in thirteen years (from 2009 to 2022), aromatic organic molecules and highly oriented pyrolytic graphite (HOPG) as models have been proven to be a reasonable strategy that could help us deeply understand the real nature of carbon catalysis.

In this Review, we summarize the extensive efforts on mechanistic understanding of metal-free carbon catalysis using model catalysts. The main content includes: (i) the fungibility between organic molecule models and graphitic carbon materials; (ii) the real contribution of each alien species, defect, and edge configuration to a series of fundamentally important reactions, such as selective thermocatalytic oxidation reaction, dehydrogenation reaction, electrocatalytic ORR and OER; (iii) the uncovering of detailed reaction mechanisms and precise structure–activity relationships of these catalytic reactions as well as unexpected rate-determining steps (RDS) at a molecular level and (iv) the specific perspective of model catalysts in catalysis. In summary, using aromatic organic molecules with designed structure and size together with bulk HOPG as models is a promising methodology to provide convinced catalytic mechanisms ranging from thermocatalysis to electrocatalysis. Thus, we suppose this Review would attract more and more attention to developing model materials in catalysis and extend avenues for catalytic studies.

## Fundamental Organic Molecule Models

**Fig. 1:**
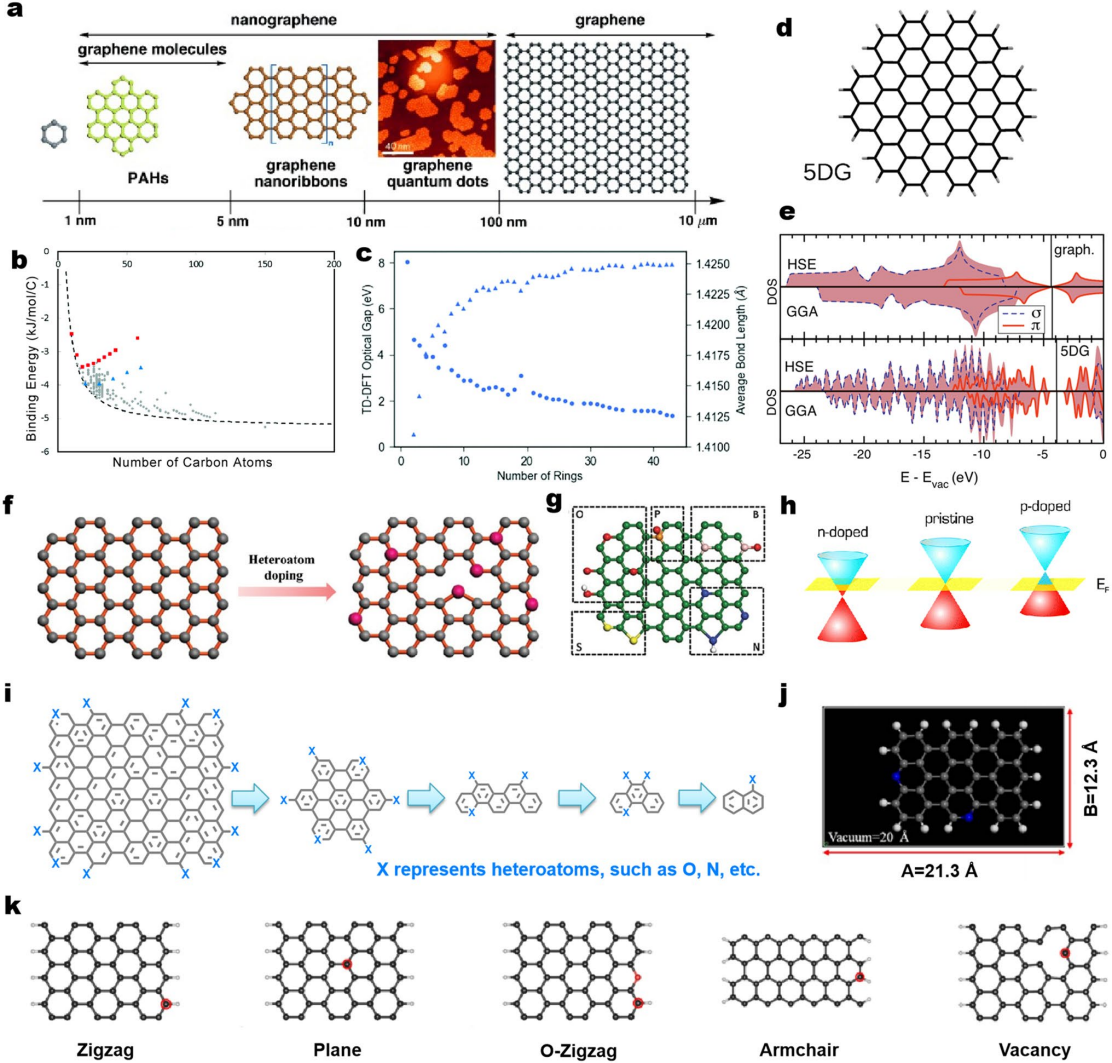
**a** Schematic illustration of graphene terminology defined according to their size scale. Graphene nanomolecules are a subset of graphene with a size between 1 and 5 nm. Nanographene units are graphene fragments with diameters of < 100 nm, while graphene should exceed 100 nm in both directions. Reprinted with permission from Ref. [49]. Copyright 2012 Wiley–VCH. **b** Binding energy as a function of number of carbon atoms in the monomer for the complete set of benzenoid molecules studied. The dashed line depicts the empirical binding energy relationship. **c** TD-DFT optical gap (circles) and average bond length (triangles) as a function of number of rings for the circular catenation PAH series. Reprinted with permission from Ref. [52]. Copyright 2017 Royal Society of Chemistry. **d**, **e** Comparison of the density of states (DOS) for large PAHs 5DG with graphene, calculated within range-separated hybrid functional (HSE) (top) and, generalized gradient approximation (GGA) (bottom). The blue dashed and red solid lines represent *π* and *σ* states respectively. The straight line indicates the Fermi energy level. Reprinted with permission from Ref. [54]. Copyright 2015 Elsevier. **f** Heteroatom doping process on graphene. **g** Schematic representation of the model of different heteroatom (B, N, O, S, and P)-doped graphene. Here, green, pink, blue, red, yellow, and orange spheres stand for C, B, N, O, S, and P atoms, respectively. Reprinted with permission from Ref. [55]. Copyright 2019 Wiley–VCH. **h** Band structure of graphene showing *p*- and *n*-type doping regarding the Fermi level. Reprinted with permission from Ref. [56]. Copyright 2016 Royal Society of Chemistry. **i** Fragmentation of doped graphene into small heteroatoms-doped PAH molecules. **j** The common theoretical model regarding N-doped carbon-based structures. The sizes of vacuum containing unit cell are about 2 nm. Blue spheres represent N atoms. Reprinted with permission from Ref. [57]. Copyright 2019 Nature Publishing Group. **k** The common theoretical models regarding carbon-based structures involving zigzag, plane, O-containing zigzag, armchair, and vacancy configurations from Ref. [58]. The number of used carbon atoms in theoretical models ranges from 22 to about 100. All the edge carbon atoms are terminated with hydrogen atoms. (Color figure online)

First, the fundamental of aromatic organic molecules was introduced. A benzene molecule is composed of six carbon atoms joined in a planar ring with one hydrogen atom attached to each, and it is classed as a hydrocarbon as it contains only carbon and hydrogen atoms. Its size is approximately 0.58 nm. As a kind of typical organic molecule, PAH is a common name given to aromatic hydrocarbons which contain more than two unsubstituted fused benzene rings (Fig. 1a) [49]. Based on the chemical nomenclature of fused PAHs recognized by the 1957 IUPAC rules, it is reasonable to define the large PAHs having sizes of 1–5 nm as graphene molecules [50]. In other words, PAHs consisting of three abreast fused benzene rings (~ 1 nm) could be roughly named graphene molecules. Nanographene can be a graphene fragment ranging from 1 to 100 nm in size. Once the size of the hexagonal *sp*^2^ carbon network exceeds 100 nm, they can be directly regarded as graphene [49, 51]. Oppositely, graphene can be structurally broken down into PAHs and eventually exist in the form of benzene molecule. Different types of carbon-catalyzed reactions have different requirements for the size of the conjugated system. Adkins et al*.* evaluated a large number of graphene molecules of different sizes [52]. Theoretical calculations and statistical data indicate that as the conjugated carbon number of PAHs increases from 6 to 20, the empirical binding energy of PAH dimers rises abruptly from 1 to 2 kJ mol^−1^ to approximately 4 kJ mol^−1^ (Fig. 1b). When the number of conjugated carbon atoms surpasses 50, the increment of binding energy slows down gradually, and eventually approaches the exfoliation energy of graphene at 5 kJ mol^−1^. For conjugated systems with a similar number of carbon atoms, binding energies increase with an increase in the percent circularity. A similar trend was observed for optical bandgaps based on theoretical calculations (Fig. 1c). On the other hand, considering the cost of synthesis and calculation, PAHs containing approximately 20–50 conjugated carbon atoms are suitable as model catalyst molecules for experimental and theoretical calculation studies. Additionally, in some carbon-catalyzed dehydrogenation coupling reactions [53], the catalytic activity is mainly from the ketocarbonyl group, and carbon is more of carrier-like role. So in some special reactions, smaller conjugated systems of model molecules are applicable.

Regardless of the edge state, graphene and PAHs show a similar density of states (DOS) of the *π* band, suggesting their similar electronic structure (Fig. 1d, e) [54, 59]. The presence of the edge states whose density of states having a sharp peak around E_F_ could give rise to localized electronic features sensitive to chemical modification. Actually, the edges of carbon-based materials, such as graphene, also perform the relatively weak localized electronic features, which makes edge sites more reactive. The doping position preference of heteroatoms is related to the type of element, bonding type, and preparation method [60]. Take the classical 900 °C thermal annealing doped graphene [61] as an example: B atom can form B–C2(–O) at the edge and BC3 in the lattice; N atoms can form pyridine N and pyrrole N at the edge, and graphite N and pyrrole N in the lattice; the O atom can be separated by the hydroxyl group, carbonyl group, lactone, ether bond at the edge, and the hydroxyl group and epoxy group in the lattice; the P atom can form P–C3(–O) in the lattice; S atoms can form C–S–C located at the edge. In addition, different synthesis methods will affect the position of doping. For instance, when graphite is exfoliated by ball milling, the broken edges will generate dangling bonds and bind to heteroatoms [55]. Moreover, depending on doping with an electron donor (*n*-doping) or acceptor (*p*-doping), different Fermi levels of heteroatom-doped carbon-based materials could be generated (Fig. 1h) [56]. Similar to graphene, doped graphene can fragment into different doped PAH molecules (Fig. 1i). These findings, therefore, give us reasons to well bridge the graphitic network structures and the known graphene molecular structure due to their similar *π*-conjugated structures and properties. Some recent studies have found that heteroatoms-doped carbon catalysts undergo unexpected structural evolution in OER and HER electrocatalytic reactions [62, 63]. Heteroatoms of N, P and Se doped carbon catalysts can be converted into high-valence oxoanions during OER process, and oxygen-abundant residues. The latter were proven to be the active site of OER. Similarly, for HER process, N dopants are hydrogenated and dissolve into the electrolyte in the form of ammonia. Consequent reconstruction of the carbon skeleton has a remarkable promotion of the HER activity. This phenomenon makes it more challenging to deeply understand the reaction mechanism of carbon-based catalysts. In addition, like other organic and carbon carriers [64, 65], nanocarbon molecules with precise structures can be further developed as supports for metal atom catalysts. Studying their catalytic performance and mechanism is very helpful in understanding the catalytic mechanism of single atom catalysts based on carbon materials.

More recently, given that the representativeness of PAHs-based nanostructures and the limitation of computing power, theoretical models of single-layer graphitic carbon consisting of limited carbon atoms, and known configurations were widely used as probes to investigate the properties of carbon-based materials in different fields. As shown in Fig. 1h, the length size (~ 2 nm) of the model consisting of 42 carbon atoms was simulated to study the contribution of N atoms during the reaction [57]. Normally, with regards to the theoretical model of carbon-based materials, a supercell of lateral size 3 × 4 or 8 × 8 was applied, and the Brillouin zone was sampled with (2 × 2 × 1 or 1 × 4 × 1 or 1 × 1 × 1) Monkhorst–Pack *k*-points (Fig. 1k) [57, 58]. Actually, when all the edge carbon atoms are terminated with hydrogen atoms in reported theoretical models, it can be found that the used models are well consistent with real PAHs-based molecules. Therefore, they can be used directly as model probe molecules to mimic various heteroatoms species and the structures of carbon catalysts, and to probe the specific activation process for catalysis. Their expectedly exclusive groups and structures can be considered as integrated active components and are very helpful to reveal the potential catalytic mechanisms. It should be noted that, due to the intrinsic physical properties (e.g., high absorbance) of carbon materials, direct spectroscopic evidence of key intermediates that can hardly be detected by in situ advanced techniques will be obtained with model molecules. For ATR–FTIR test, the thickness and uniformity of carbon materials have high requirements [66]. The signals of different functional groups are easy to overlap with each other. For XPS test, only the signal on the sample surface can be detected. The signal originated from different heteroatom bonding type can be easily hidden in the features of the other bonding type. On the other hand, the results of spectral are macroscopic information, and cannot be completely equivalent to the exact molecular structure. Therefore, the bottom-up approach to build model catalysts with accurate structures is a promising approach to study the structure–activity relationship of carbon-based electrocatalysts. Additionally, the electronic and reactive properties of active components can be tuned by extending the *π*-conjugated domains of these model molecules at a discrete molecular level. These merits enable the understanding of metal-free carbon catalysis at an unexpected level, even at a single-molecule level.

## Brief Historical Perspective on the Application of Model Catalysts

Metal-free carbon catalysis can be traced back to about one hundred years ago when Rideal and Wright reported that charcoal could catalyze the aerobic oxidation of oxalic acid [67]. Since then, tremendous works regarding carbon catalysis have been reported, and various advanced techniques involving in situ (or operando) characterizations have been developed to understand catalytic natures. However, the underlying nature is still controversial, as some key factors which could influence the whole catalytic systems are still difficult to be completely ruled out. In 2009, Prof. Schlögl and his colleagues introduced a model catalyst strategy for the first time by using fused phenanthrenequinone with exclusive C=O group to identify the active sites toward ethylbenzene (EB) oxidative dehydrogenation (ODH) reaction [68]. In the next ten years, PAHs and O- or N-doped PAHs were used as model molecules toward various typically thermocatalytic and electrocatalytic reactions. For example, nitrobenzene reduction, alcohol oxidation, ORR, and OER are carefully studied by using various PAHs-based molecules with designed edge configurations or alien groups, and some striking achievements are made, suggesting that the introduction of aromatic organic molecules as models is feasible to carry out the catalytic reactions and get insight into mechanisms. The roles of conjugated structure, spatial structure of model molecules, and different heteroatom species were emphasized and were classified. Moreover, using doped bulk catalyst, such as HOPG, as model catalyst was also developed. In the following two parts, we will discuss the highlights and contributions of molecule model and bulk model catalysts in different reactions.

## Molecule Models in Thermocatalytic Reactions

**Fig. 2:**
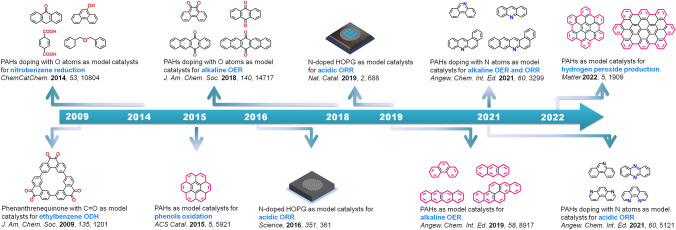
Development of model catalysts in metal-free carbon catalysis. Here, ODH stands for oxidative dehydrogenation reactions

Thermal-driven catalysis (thermocatalysis), mainly including liquid-phase and gas-phase reactions, involves wide scope and is relatively easy to realize practical large-scale applications. It generally requires the used catalysts to have good thermal conductivity and thermal stability during the reactions. Metal-free carbon materials possess abundant edge heteroatom species (e.g., C=O, C–O), surface chemical structures (e.g., vacancy, five-membered ring), stable structure, and good physical property that are capable of manipulating the fast adsorption process of reactants and desorption process of desired products, thus enabling promising applications in different industrial catalytic reactions [37, 38]. To analyze the actual contribution of the active site to the reaction, several works have employed model molecules containing carbonyl groups and model molecules containing pyridine nitrogen as catalysts for different catalytic dehydrogenation and catalytic oxidation reactions. Depending on the reaction media, the gas-phase reaction requires better thermal stability for carbon itself and higher demands for operating equipment than those of liquid-phase reactions. This section will be discussed on the basis of two reaction classifications (Fig. 2).

### Gas-Phase Oxidative Dehydrogenation Reactions

Ethylbenzene (EB) oxidative dehydrogenation (ODH) is an energy-saving candidate for a typical industrial production of styrene which is an important organic precursor for the synthesis of polyethylene and other organic compounds. Usually, the catalysts used in ODH reactions are transition metals and metal oxides, such as Fe_2_O_3_, Pt-, Cr_2_O_3_-, V_2_O_5_-, MoO_*x*_-, Ga_2_O_3_-based catalysts, and so on [20]. Recently, carbon materials including nanodiamond, onion-like carbon, cokes, activated carbons, and graphite are as efficient as metal catalysts [69, 70]. The proposed results indicated that nanocarbon with a graphitic structure provides high activity over a long period due to the controlled homogeneity of the support structure and the chemical uniformity of the active sites [37]. With assistance of ex situ and quasi in situ characterizations, the structures of active sites had been proposed to consist of diketone- and/or ketone-like groups, which are initially generated during the synthesis and maintained in the form of their oxidation states by connecting with the reactant O_2_ [71]. Mechanistic investigations reflect that breaking C–H bonds may be kinetically limited in the reaction sequences [72]. Styrene molecules desorb from the catalyst’s surface, while the produced hydrogen is oxidized to water. The precise structure of the active site, the detailed reaction mechanism, and their contribution to the reaction rate still need to be addressed.

**Fig. 3:**
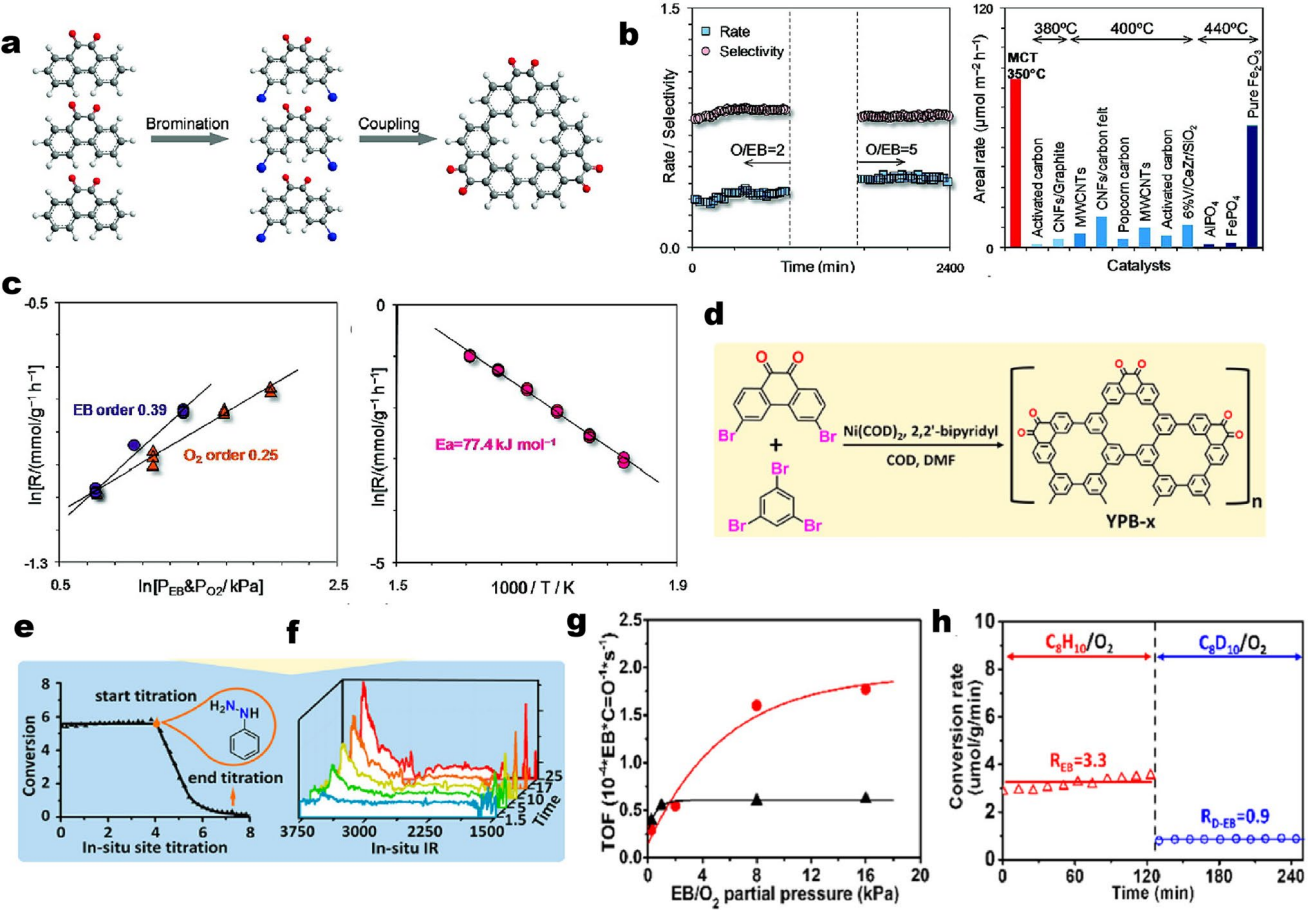
**a** Schematic synthesis of the fused phenanthrenequinone (macrocyclic trimer, MCT) model catalyst toward ethylbenzene (EB) oxidative dehydrogenation (ODH) reaction. **b** Reaction rate (mmol g^−1^ h^−1^) and styrene selectivity on MCT oligomer (left side) and comparison of activities with typically reported catalysts (right side). **c** Dependencies of reaction rate on partial pressures of each reactant (left side) and temperature (right side). Reprinted with permission from Ref. [68]. Copyright 2008 American Chemical Society. **d** Schematic drawings of the synthesis procedure for model catalysts (YPB-x). **e** EB conversion as a function of time on stream during the steady-state activity measurement and in situ titration process. **f** In situ IR spectra of model catalysts upon the introduction of EB as a function of the reaction time. **g** ODH rate as a function of EB (circle) and O_2_ (triangle) partial pressure. **h** ODH rates with C_8_H_10_(EB)/O_2_ (empty triangle) and C_8_D_10_(deuterated EB)/O_2_ (empty circle) on model catalysts. Reprinted with permission from Ref. [73]. Copyright 2017 American Chemical Society

Based on it, Zhang and co-workers introduced a model molecule approach to provide direct evidence for understanding the chemical structure of the active sites [68]. Such macrocyclic trimer (MCT) model molecules consist of fused benzene rings equipped with only C=O groups synthesized by the combinations of bromination, coupling, and recrystallization processes, as shown in Fig. 3a. Then, the performance of MCT oligomer was evaluated. Figure 3b shows the catalytic function of MCT at 350 °C along with the time-on-stream. The sample was immediately active and quickly reached a steady state, in which the reaction rate and styrene selectivity remained above 0.34 mmol g^−1^ h^−1^ and 86%, respectively. Moreover, the O/C ratio increases from 2 to 5, which will improve the reaction rate to 0.44 mmol g^−1^ h^−1^ at constant styrene selectivity (83%). CO_*x*_ was the major byproduct. A comparison in performance between MCT and typically reported catalysts showed that the activity of MCT is up to 47 times that of other catalysts including metal phosphates and oxides. Furthermore, the reaction rate (*R*) displays a positive dependency on the partial pressures of ethylbenzene and O_2_ (*P*_*i*_), revealing reaction orders of 0.39 and 0.25 for ethylbenzene and O_2_, respectively (Fig. 3c, left side). An activation energy (*E*_a_) of 77.4 kJ mol^−1^ was calculated from the slope of an Arrhenius plot (Fig. 3d, right side). The authors, therefore, proposed that all kinetic parameters are close to those determined from carbonaceous materials in previous studies, confirming the similarity of both mechanism and structure of active sites. MCT gives a real rate 5–9 times those of the nanocarbons at the same conditions. The superior activity is associated with the abundance of diketone groups for styrene formation. The nonzero reaction orders for both EB and O_2_ indicate the matched rates for the organic transformation and the oxidation of hydrogen in contrast to systems where lattice oxygen decouples the regeneration of the active site from the organic transformation. This work suggests that MCT is a suitable molecular model to study ODH catalysis. Its outstanding performance provides direct evidence and confirms the previous hypothesis that the carbon-catalyzed ODH process may be mediated by diketone- and/or ketone-like functional groups.

More recently, Guo et al*.* used a polymerization method to synthesize a new class of model molecules (Yamamoto-derived phenanthrenequinone & benzene networks, denoted as YPB) which also contained only ketonic carbonyl groups at their periphery (Fig. 3d) [73]. Importantly, the abundance of active sites (ketonic carbonyl group content from 1% to 7.4%) and the surface area (from 560 to 1450 m^2^ g^–1^) of the model molecules could be adjusted by controlling the ratio of the precursors, which is comparable to typical nanocarbon materials (normally 2%–10% oxygen content and 500–1000 m^2^ g^–1^). This team further performs EB ODH kinetic measurements with such model molecules to reveal the reaction mechanism. Based on in situ titration strategy (using phenylhydrazine), the authors found that the number of active sites from titration could sometimes be slightly lower (~ 5%) than the total amount of O species on the catalyst surface (Fig. 3e). An in situ infrared (IR) analysis provided spectroscopic evidence of the “working” configurations of the active sites in association with the redox cycle of the ketonic carbonyl-hydroxyl pairs (Fig. 3f). Additionally, the ODH rate increased monotonically with increasing EB partial pressure, while it reached constant values at O_2_ pressures higher than 1 kPa (Fig. 3g), suggesting that O_2_ has limited influence on reaction rates in this regime. The observed kinetic behaviors on EB and O_2_ partial pressure are consistent with kinetic isotope effect (KIE) results (Fig. 3h). The latter’s findings imply that the reaction is kinetically controlled by the C–H activation process, namely, the breaking of C–H bond is the RDS over a relatively wide alkane/O_2_ ratio range. Both of Zhang’s and Guo’s work suggests that using model molecules is feasible to study active sites and reaction mechanisms toward gas-phase reactions, especially for EB ODH.

### Liquid-Phase Oxidation and Reduction Reactions

**Fig. 4:**
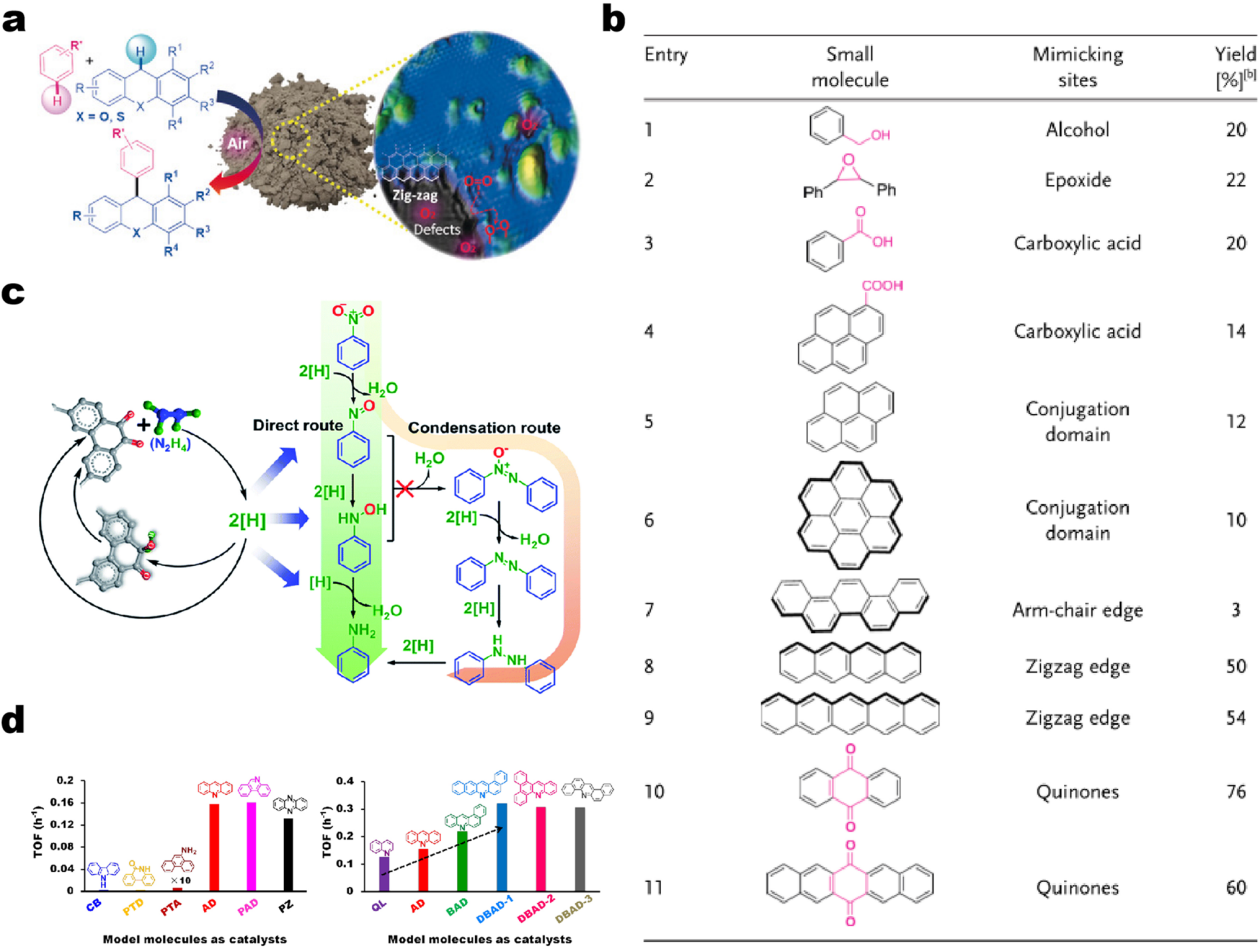
**a** Cross-dehydrogenative coupling of xanthene (or thioxanthene) and arenes catalyzed by carbon materials. Mechanistic studies suggest that, compared to oxygenated groups, zigzag edge sites play a greater role in carbocatalysis than previously supposed. **b** Performance evaluation of small molecules with various O species and edge configurations aiming to reveal the possible active sites. Reprinted with permission from Ref. [53]. Copyright 2018 Wiley–VCH. **c** Schematic drawings of model molecules with exclusive C=O group catalyzed nitro compounds reduction process. Reprinted with permission from Ref. [76]. Copyright 2016 Royal Society of Chemistry. **d** TOF values of model molecules with various N species (e.g., pyrrole, pyridine, lactam), gradually extended *π*-conjugated structure, and different structural configurations used as catalysts toward aerobic alcohol oxidation. Reprinted with permission from Ref. [77]. Copyright 2019 American Chemical Society

Su’s group extended the scope of metal-free carbon catalytic reactions in which CH–CH-type cross-coupling of xanthenes with arenes is developed (18 examples, Fig. 4a) under solvent-free and air conditions [53]. The synergistic catalytic effect of carbon catalysts with TsOH·H_2_O provided the best results (85% NMR yield). The recovered carbon catalysts could be recycled and maintained their high catalytic activity (68%) up to the fifth run. Moreover, the relationships between the type of functional groups present and the specific activity of the carbon catalysts were well established. The authors suggested that not all oxygen functional groups have vital roles in this catalytic system with some control experiments. Scanning tunneling microscope studies revealed that thermally processed catalysts possess a high density of zigzag edges around defective sites. Large PAHs, classified as nanographene, have two main edge structures: zigzag and armchair edges. Theoretical calculations showed that armchair edges exhibit higher aromaticity and stability compared to zigzag edges [74]. Whereas local density of states (LDOS) and isovalue of frontier orbitals tend to be more distributed at the zigzag edges, which makes them behave like free radicals [54, 75]. These properties were supported by further experiments. By comparing with the activity of different model molecules, the real contributions of alien species and edge configurations were found (Fig. 4b). Model molecules such as benzyl alcohol, 2,3-diphenyloxirane, benzoic acid, and 1-pyrenecarboxylic acid which mimic the hydroxy, epoxide and carboxyl groups, showed low catalytic activity (Fig. 4b, entries 1–4). PAHs such as pyrene, coronene, and picene, which are characterized by conjugated structures and armchair edges, obtained less than 12% yield (Fig. 4b, entries 5–7), whereas tetracene and pentacene with zigzag-edged counterparts exhibit higher reactivity. Anthraquinone consisting of both the zigzag edges and the C=O species afforded the best performance among all the tested small-molecule models. This result echoes the previous observation that the reactivities of carbon materials are linked to C=O species (Fig. 4b, entries 10–11). These findings provide evidence for revealing the contribution of both the quinone-type functionalities and the zigzag edges in carbon catalysts to the coupling reaction. Mechanistic studies further suggest that, compared to oxygenated groups, zigzag edge sites play a greater role in carbocatalysis than previously supposed.

To determine the possible catalytic reaction pathways of nitro compounds reduction process in carbon catalytic system, YPB with only C=O groups and defined chemical structures as model molecules were used [76]. With regards to two typical reaction pathways including the direct and the condensation routes (Fig. 4c), the authors believed that the direct route may dominate the reaction pathway as less than 1% of byproducts (azobenzene and azoxybenzene) is detected in the model molecules-catalyzed nitro reduction process. This finding is also supported by the first-order kinetics, which is consistent with the reaction order of RDS of the direct route (forming nitrosobenzene). Together with IR measurements, product distribution, and kinetic analysis of the catalytic reaction, the authors proposed a plausible mechanism for carbon-catalyzed aromatic nitro compounds reduction. The C=O groups serving as active sites could activate and interact with hydrazine forming active reducing species ([H]), which are further capable of reducing nitro into the desired aniline product. The nitro reduction follows the direct route through a sequential hydrogenation process with aniline and H_2_O as final products.

More recently, N functionalized aromatic molecules had been applied to study the real contribution of each common N species (e.g., pyrrole, pyridine, amidogen, lactam) and to establish a precise structure–function relationship for aerobic alcohol oxidation (Fig. 4d) [77]. The results indicate that pyridinic N species play an unexpected role in catalytic reactions. The nearly linear structure–function relationship of the active components can be established by delicately controlling their longitudinal extension (*π*-conjugated structures), local environment, and distribution density. Moreover, pyridinic N groups located at zigzag and armchair edge configurations show similar catalytic performance, and each pyridinic N is demonstrated experimentally to contribute independently to the probe reaction (Fig. 4d, right side). The activation process of the α–H of –CH_2_OH over both bulk and model catalysts is clarified to be an RDS of the catalytic reaction using KIE. Neighboring carbon atoms in pyridinic N species are responsible for facilitating the RDS process. These findings give important evidence at a real molecular level for the identification of real active sites with metal-free carbon as catalysts in alcohol oxidation. However, up to now, direct comparative studies of pyridine nitrogen, carbonyl groups, and other functional groups for catalytic dehydrogenation and catalytic oxidation are still lacking.

## Model Catalysts in Electrocatalytic Reactions

The ongoing energy and chemistry transition characterized by the progressive electrification and substitution of raw materials with alternative sources to decrease fossil fuel use has spurred the development of nanomaterials to enhance the reaction performance in electrocatalysis. Metal-free carbons offer valuable alternatives as electrode materials with low cost, abundant structural varieties, tailorable surface chemistry, and stability. Heteroatom-modified nanostructured carbons with tunable surfaces further show promising opportunities for realizing sustainable applications [40]. With the rapid development of material design and catalytic performance of metal-free carbon in electrocatalytic reactions, the mechanistic understandings of metal-free carbon catalysis also make some striking achievements with specific advanced techniques and strategies. This section will focus on overall oxygen electrocatalytic reactions including ORR and OER revealed by model catalysts. Carbon-based catalysts with precise molecular structures will be highlighted for electrocatalytic mechanism studies. The catalytic performance of PAHs doped with main group elements B, N, O, etc. varies significantly with the substitution position of heteroatoms and the bonding mode. These studies will help us to better understand the structure–activity relationships of carbon-based catalysts.

### ORR

#### Four-Electron Process

**Fig. 5:**
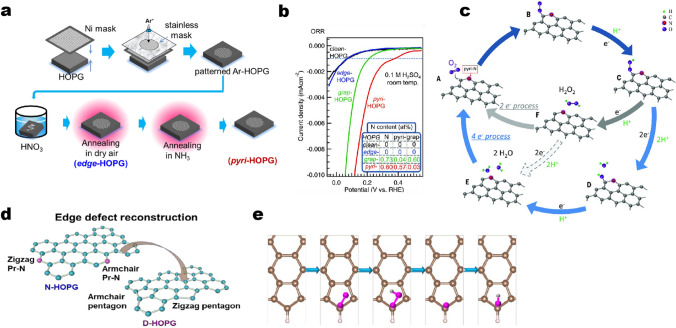
**a** Schematic illustration for the preparation of edge-HOPG and pyri-HOPG model catalysts. **b** Performance evaluation of model catalysts toward acidic ORR. **c** Schematic reaction pathway for ORR on N-doped carbon materials. Reprinted with permission from Ref. [78]. Copyright 2016 American Association for the Advancement of Science. **d** Illustration of edge defect reconstruction aiming to remove original N species at elevated temperature. **e** The ORR pathway on pentagon edge configuration under acidic condition. Reprinted with permission from Ref. [57]. Copyright 2019 Nature Publishing Group

Guo et al*.* used HOPG with well-defined *π* conjugation as a precursor, followed by an Ar^+^ etching approach to obtain doped HOPG with well-controlled doping of N species (Fig. 5a), which can be used as bulk model catalysts to understand the actual role of N species and to elucidate the active sites [78]. The activity evaluation results indicate that the pyridinic N species dominate ORR process, and are supposed to be active sites (Fig. 5b). Carbon dioxide adsorption experiments indicated that pyridinic N also creates Lewis basic sites. A nitrogen atom bound to two carbons formed an active catalyst site with an activity rivaling that of N-doped graphene catalysts. Thus, the ORR active sites in N-doped carbon materials are carbon atoms with Lewis basicity next to pyridinic N. Moreover, the authors proposed a plausible reaction pathway. O_2_ is first adsorbed at the carbon atom next to the pyridinic N, followed by protonation of the adsorbed O_2_. Two pathways are then possibly involved. One is the 4 e^−^ mechanism occurring at a single site, and the other is the (2 + 2) e^−^ mechanism, which does not often happen at a single site. With regard to the 4 e^−^ mechanism, the other two protons attach to the two O atoms, leading to breakage of the O–OH bond and formation of OH species (“D” in Fig. 5c). The additional proton then reacts with the adsorbed OH to form H_2_O (“E” in Fig. 5c). In the (2 + 2) e^−^ pathway, H_2_O_2_ is formed by reaction of the adsorbed OOH species with another proton (“F” in Fig. 5c), followed by readsorption of H_2_O_2_ and its reduction by two protons to generate H_2_O. The authors believed that both 4 e^−^ and (2 + 2) e^−^ mechanisms involve the adsorption of O_2_ molecules, which is the initial step of the ORR, suggesting the critical role of the carbon atom next to the pyridinic N. More recently, a different reaction mechanism concerning active sites has been reported by using almost the same synthetic method [57]. By means of additionally evaluated temperature at N_2_ atmosphere, edge defects of model catalysts were reconstructed along with the removal of original N species and in situ formation of a new pentagon edge configuration (Fig. 5d). Combined work function analyses and macro/micro-electrochemical performance measurements, the pentagon defects in model catalysts served as major active sites for acidic ORR are raised. The authors believed that the contribution of pentagon defects is higher than that of common pyridinic N. The reaction mechanism taking place at pentagon defects generally involves four H^+^ and electron transfer steps (Fig. 5e): (i) the adsorbed O_2_ transfers into OOH*; (ii) desorption of H_2_O and formation of O*; (iii) OH* is formed and (iv) the OH* further associates with a protic H and an electron to generate H_2_O.

**Fig. 6:**
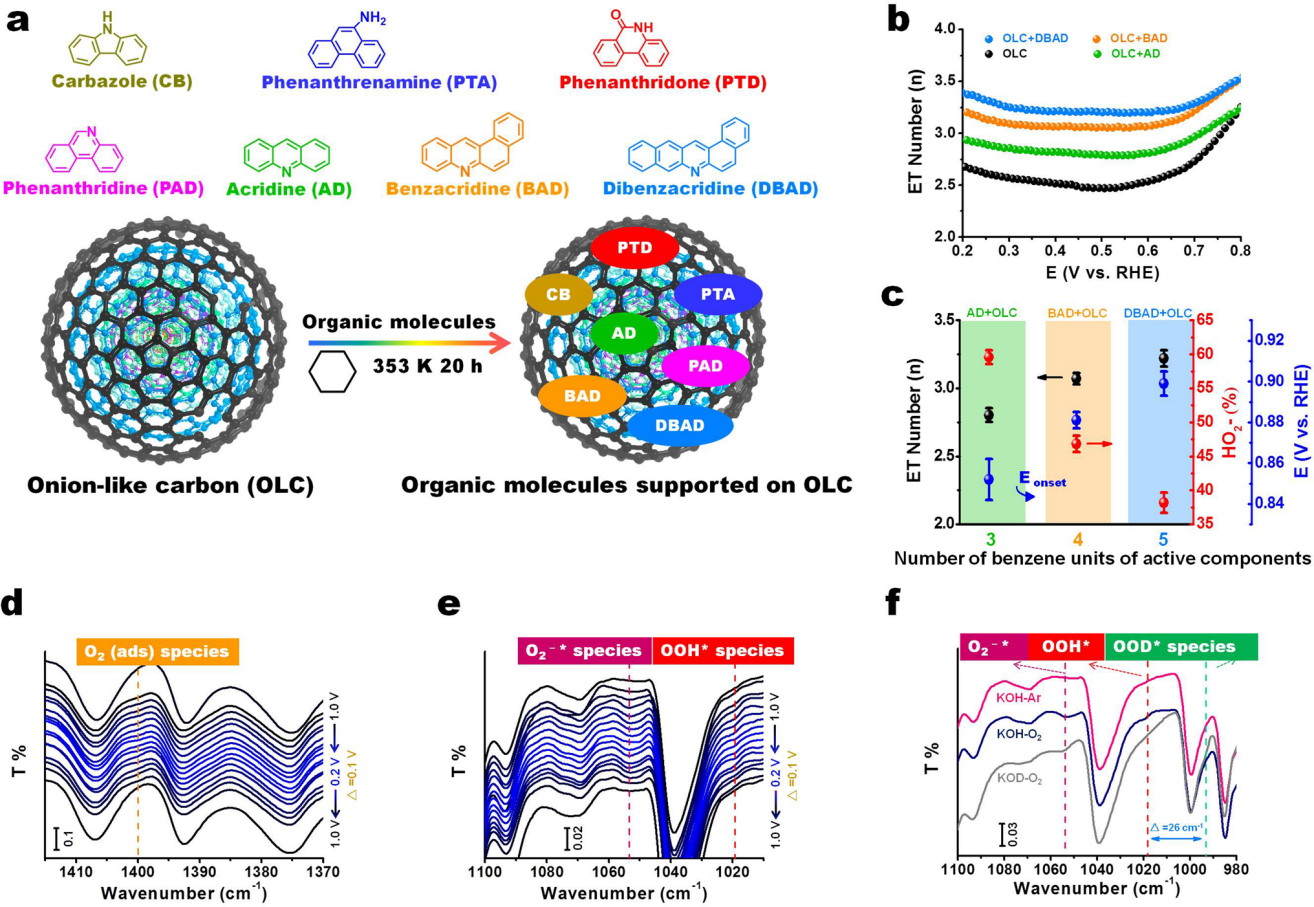
**a** Structures of different model molecules with isolated N configurations (including pyrrolic, amine, lactam and pyridinic N) and the preparation of model molecules-supported on onion-like carbon (OLC) catalytic system using a solvothermal method. **b** Electron transfer (ET) numbers of three supported catalysts with isolated pyridinic N species based on rotating ring-disk electrode measurements. **c** The relationships between the number of benzene units of model molecules with single pyridinic N species and ET number, HO_2_^−^ or onset potential (*E*_onset_). **d**–**f** In situ ATR-IR spectra coupling with isotopic labeling experiments for identifying and monitoring dynamic evolution of the involved intermediate oxygen species including adsorbed O_2_, O_2_^−^* and OOH* species. Reprinted with permission from Ref. [79]. Copyright 2021 Wiley–VCH

Given that the recent mechanistic understanding of active sites, adsorbed intermediate products, and rate-determining steps (RDS) of N-doped carbon catalysts in ORR are still rife with controversy, Lin and his colleagues applied several functionalized molecules with isolated N configurations as active models to address the above scientific issues [79]. The onion-like carbon (OLC) was used as support to disperse model molecules (Fig. 6a). Compared with the catalytic performance of different model molecules, it points out the fact that pyridinic N species play a crucial role for ORR over a wide pH range. The structure–function relationship of the active models can be established by delicately controlling their longitudinal extension (*p*-conjugated structures) and edge configurations. It can be concluded that pyridinic N species are prone to facilitate the ORR process by a 4 e^−^-like pathway (Fig. 6b, c). Furthermore, the location at edge zigzag or armchair positions of pyridinic N does not obviously affect the catalytic performance. Some reversible dynamic evolution behaviors concerning different intermediates are monitored with in situ ATR-IR spectra (Fig. 6d–f). The authors proposed that adsorbed O_2_ molecules, OOH* and O_2_* species can be identified, and are involved as the key intermediates in ORR processes, which was corroborated by isotopic labeling studies. The formation of *OOH species from O_2_^−^* (O_2_^−^* + H_2_O → OOH* + OH^−^) as a possible RDS during the ORR process was suggested.

**Fig. 7:**
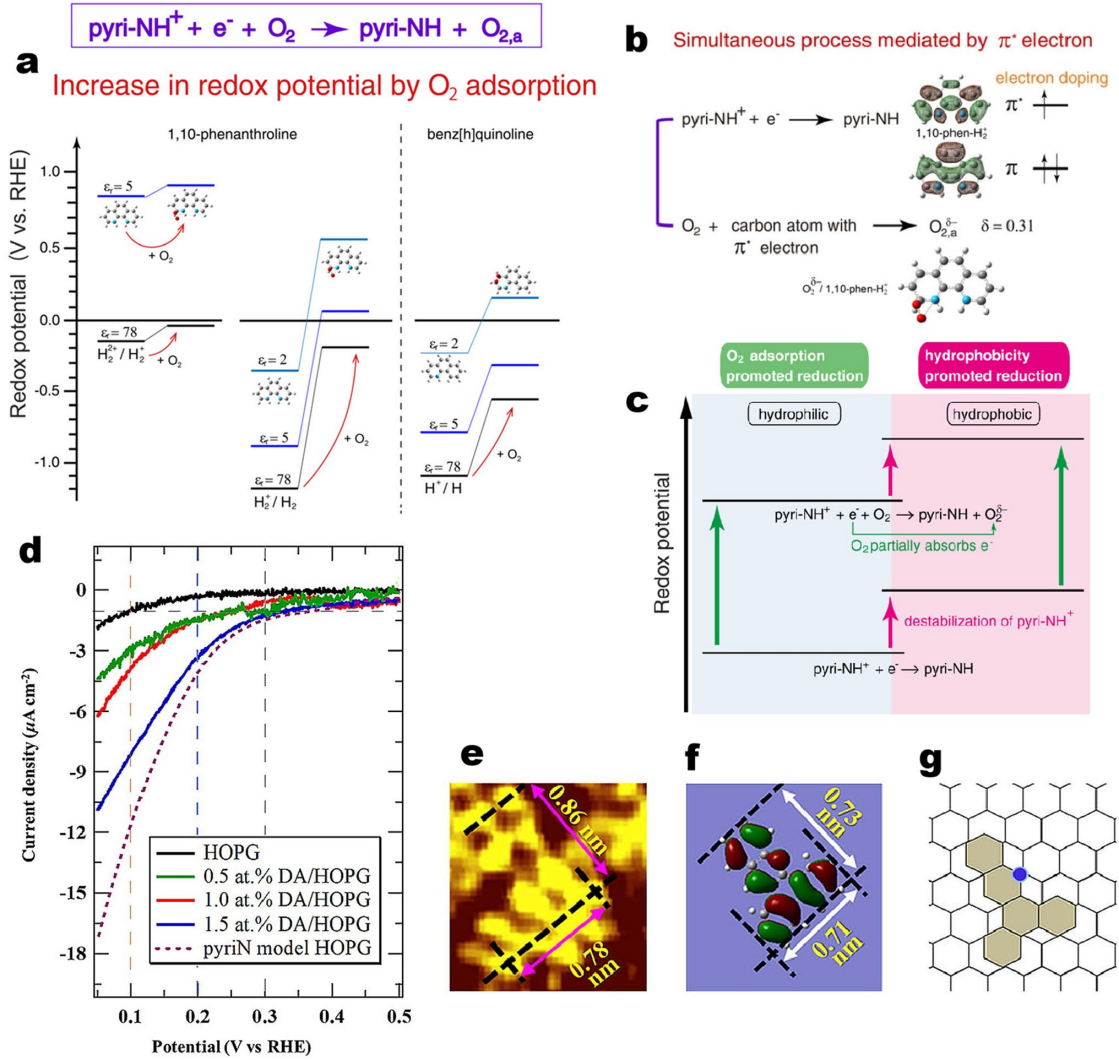
**a** Calculated redox potentials of pyri-NH^+^/pyri-NH with and without O_2_ adsorption for 1,10-phenanthroline and benz[h]quinoline in water at different relative permittivities. **b** π* electron-mediated simultaneous reduction, where electron is supplied into π*orbital of 1,10-phenanthroline, which was used for O_2_ energy gain due to the adsorption energy of O_2_. **c** Promotional effects of O_2_ conditions on the reduction of pyri-NH^+^. Reprinted with permission from Ref. [80]. Copyright 2021 Wiley–VCH. **d** ORR results for various concentrations of DA. The ORR results for pristine HOPG and the pyridinic-nitrogen HOPG model catalyst (pyri N model HOPG) are also shown. Electrolyte conditions: 0.1 M H_2_SO_4_, room temperature. **e** Magnified STM image of the DA molecule on HOPG. **f** HOMO of isolated DA. **g** DA molecule in the model structure in HOPG. Reprinted with permission from Ref [81]

Meanwhile, Takeyasu et al. further elaborated the reaction pathways of pyridinic N species also by using N functionalized phenanthrene and anthracene as model molecules [80]. The results indicate that the 1,10-phenanthroline with two pyridinic N atoms at armchair edges shows the highest activity in the presence of carbon black support. Under acidic conditions, pyridinic N atoms of 1,10-phenanthroline could be protonated to form pyridinium ions (pyri-NH^+^). In O_2_-saturated electrolytes, one species was reduced to pyri-NH upon the application of a potential. This behavior was ascribed to electrochemical reduction of pyri-NH^+^ occurring simultaneously with the thermal adsorption of O_2_, as supported by DFT calculations (Fig. 7a). The reduction of pyri-NH^+^ coupled with O_2_ adsorption was explained by supply of an electron into the π* orbital of 1,10-phen-H_2_^2+^ (Fig. 7b). The adsorbed O_2_ is partially negatively charged (Fig. 7b). Furthermore, the pyri-NH^+^ coupled reduction was promoted by hydrophobic environment (Fig. 7c).

Shibuya et al. reported a preparation of a model catalyst bydropping a solution of dibenz[a,c]acridine (DA) molecules onto a highly oriented pyrolytic graphite (HOPG) surface [81]. As shown in Fig. 7e–g, the scanning tunneling microscope (STM) image resembles the calculated HOMO of the DA molecule in terms of size and shapes of the orbitals, which indicates that the DA molecules are adsorbed on the HOPG surface with a flat configuration. The current densities for the DA/HOPG electrodes increased linearly with the concentration of DA on HOPG at potentials of 0.1, 0.2, and 0.3 V (vs. RHE) (Fig. 7d), this indicates that the DA molecules provide active sites for the ORR.

**Fig. 8:**
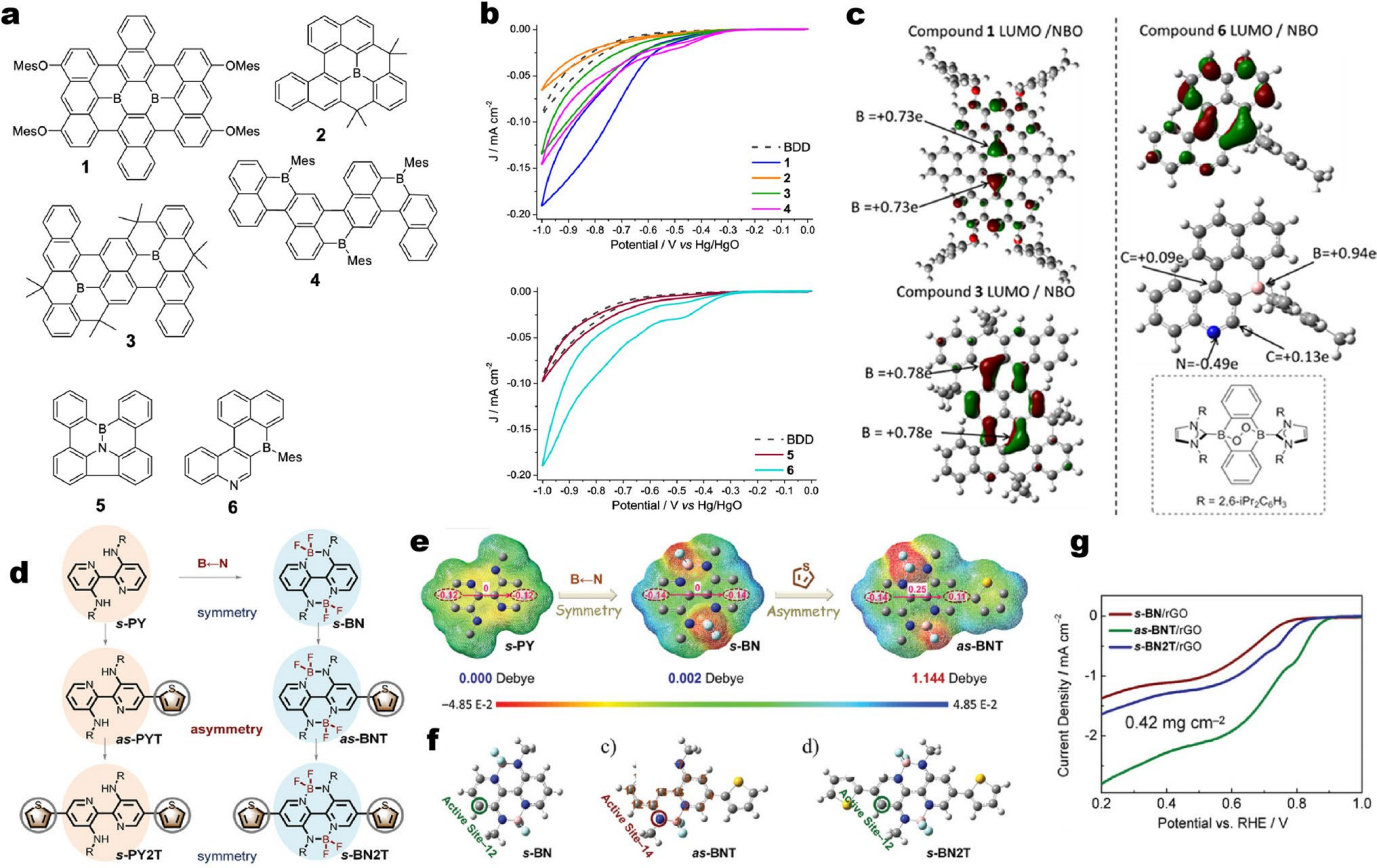
**a** Well-defined doped PAHs used herein and the comparison compounds quinone A and perylene (compound B). Mes = mesityl, a boron “protecting group”. **b** CVs of **1** − **4** and **5** − **6** on a BDD electrode in O_2_-saturated 0.1 M KOH (aq) at a scan rate of 50 mV s^−1^. **c** LUMO (at isosurface value = 0.04) and select NBO charges for compounds **1**, **3**, and **6**. The inset shows the product from the reaction of a NHC-stabilized 9,10-dibora-anthrene with O_2_ (NHC = N-heterocyclic carbene). Reprinted with permission from Ref. [82]. Copyright 2019 American Chemical Society. **d** The chemical structures of symmetric (*s*-PY, *s*-PY2T, *s*-BN, and *s*-BN2T) and asymmetric (*as*-PYT and *as*-BNT) organic molecular catalysts. **e** The electrostatic potentials and dipole moments of *s*-PY, *s*-BN, and *as*-BNT. **f** The optimized molecular structures of *s*-BN, *as*-BNT and *s*-BN2T models. **g** LSV curves of *s*-BN/rGO, *as*-BNT/rGO, and *s*-BN2T/rGO. Reprinted with permission from Ref. [83]. Copyright 2022 Wiley–VCH

Kahan et al*.* studied a series of B- and B, N-doped model molecular ORR catalysts (Fig. 7a) [82]. Six model catalysts were synthesized by bottom-up method and then were loaded on the surface of boron-doped diamond (BDD) electrode. It was found that compound **1** and **6** have the best activity and compound **2** and **5** show no activity towards ORR (Fig. 8b). Their LUMO energies were analyzed by DFT calculation (Fig. 8c). The authors speculated that the following two factors have important effects on catalytic activity: One is the low LUMO energy of model compounds. The second is B, N dopants with no direct bonding, which is conducive to the generation of two electrophilic active sites in close proximity for bidentate O_2_ binding. Recently, Wang et al*.* investigated the effect of asymmetric regulation of B–N bonds for ORR, in which different conjugated organic molecules with B–N coordination bonds and thiophene rings are carefully introduced (Fig. 8d) [83]. The model catalyst molecules were dispersed over reduced graphene oxide (rGO) aiming to increase the specific surface area. The symmetry, charge distribution and dipole moment of the molecules can be changed by introducing S heterocyclic rings (Fig. 8e). For example, the asymmetric *as*-BNT exhibits the dipole moment of 1.144 Debye, which is larger than its symmetric counterparts (0.002 Debye for both *s*-BN and *s*-BN2T), indicating that these asymmetric molecules increase the backbone polarity and benefit non-uniform charge distribution of molecular skeletons. Moreover, asymmetric regulation also significantly changes the catalytic activity site from *sp*^2^ C atoms (site-12) of the symmetric *s*-BN and *s*-BN2T to amino-N atom (site-14) of the asymmetric *as*-BNT (Fig. 8f). The latter has the best ORR activity (Fig. 8g). The results of DFT calculations suggest that the asymmetric structure resulted in the transfer of the active site from C atom to N atom. A similar phenomenon was observed for some conjugated polymers [84].

#### Two-Electron Process

Electrosynthesis via two-electron ORR with doped carbon materials is one of the most feasible ways for on-demand production of H_2_O_2_ [85, 86]. Various heteroatoms species attached to carbon edges are proposed to be active evidenced by impressively experimental characterizations and theoretical calculations. However, the real contributions of intrinsic carbon edges (heteroatoms-free) to electrosynthesis H_2_O_2_ have been lacking experimental insights as it is hard to study them in isolation from the coexistence of heteroatoms species. Therefore, finding an effective method to determine the explicit function of each common carbon edge at a molecular level is of critical importance for revealing the concealed reaction mechanism toward electrochemical H_2_O_2_ production.

**Fig. 9:**
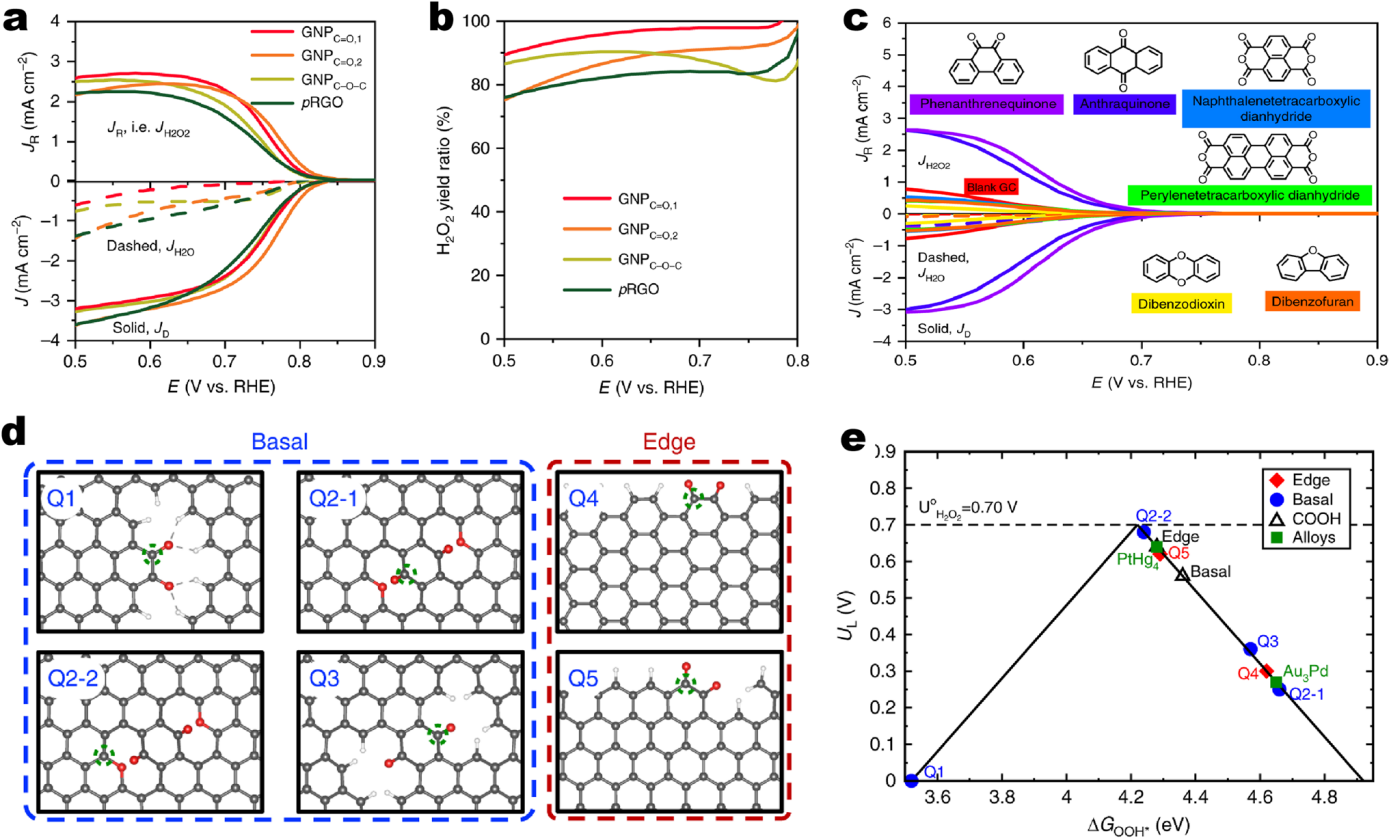
The performance characterization and mechanism study of electrosynthesis H_2_O_2_ on O-enriched graphitic nanoplatelets (GNP). **a**, **b** The activity and yield of H_2_O_2_, respectively. **c** The electrosynthesis H_2_O_2_ performance of model molecules with different O groups. **d** Theoretical structures of different oxygenated groups at basal and edge sites. **e** Theoretical H_2_O_2_ activity volcano plot. Horizontal dashed line corresponds to the thermodynamic equilibrium potential for H_2_O_2_ electrosynthesis (*U*_0_ = 0.70 V). Reprinted with permission from Ref. [87]. Copyright 2020 Springer Nature

Han et al*.* adopted a pre-activated method to decorate the dangled edges of graphitic nanoplatelets (GNP) with targeted groups (ether, carboxyl and quinone) [87]. With the careful performance evaluation (Fig. 9a), the results confirmed a new class of quinone-edged groups, which exhibited higher selectivity than previously reported oxygenated groups with similar onset potential. The quinone-enriched samples exhibited a H_2_O_2_ yield ratio of 97.8% at 0.75 V (Fig. 9b). The results were further verified using stand-lone molecular chemistry. Molecules with quinone, carboxylic acid, and etheric ring groups were chosen as the model catalysts, such as phenanthrenequinone, anthraquinone, naphthalenetetracarboxylic dianhydride, perylenetetracarboxylic dianhydride, dibenzodioxin, and dibenzofuran. The polarization curves are shown in Fig. 9c. Except for phenanthrenequinone and anthraquinone, the other four molecules did not show activity towards electrosynthesis H_2_O_2_; the activity was inferior to blank glass carbon (GC). The phenanthrenequinone was superior to anthraquinone in both the J_K,H2O2_ (0.7 vs. 0.5 mA cm^−2^ at 0.65 V) and Tafel slope results (45 vs. 48 mV dec^−1^). These molecular chemistry results further confirm that the quinones are the active sites. Additionally, the common DFT calculations was used to study the different possible quinone groups on the edge and basal planes (Fig. 9d, e). The free energy results of quinone functional groups at different sites were summarized in an activity volcano plot. The quinone functional groups on the edge (Q-edge 5) were comparable to the PtHg4 catalysts, and the Q-basal 2–2 displayed the highest activity. Moreover, the authors proposed that the formation of quinone functional groups on the edge seems more feasible than in the basal area, because the formation of Q-Basal groups significantly interrupts the *sp*^2^ network and requires a lot of energy input.

**Fig. 10:**
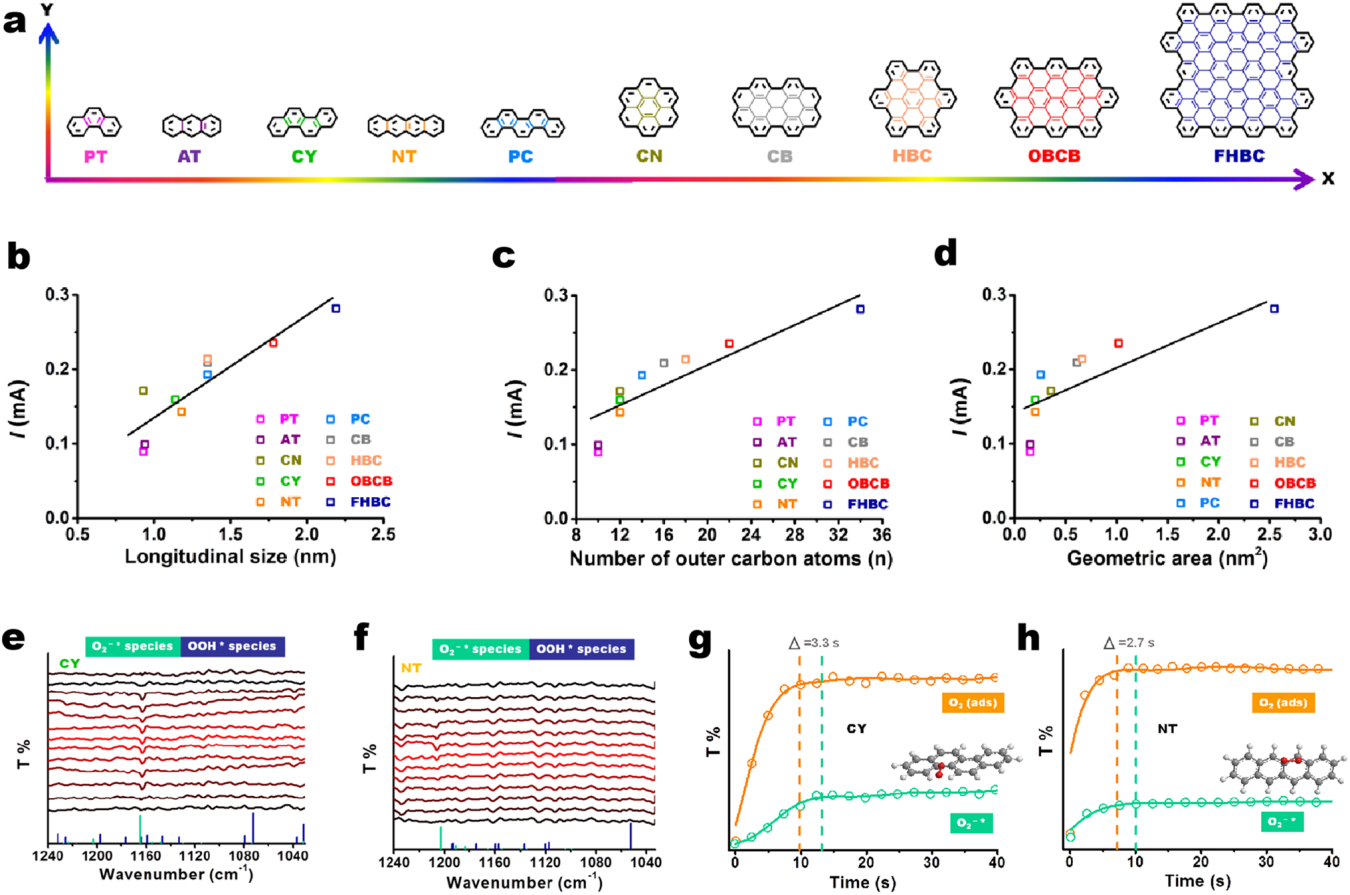
Structure/size/area-function relationships and electrochemical performance of PAH catalysts for electrosynthesis H_2_O_2_. **a** Trends of gradient structures of PAH catalysts. **b**–**d** The correlations among the longitudinal sizes, the number of exposed outer carbon atoms and the geometric areas of PAHs and the disk current at 0.6 V_RHE_. **e**, **f** In situ ATR-IR spectra for monitoring dynamic evolution of the involved intermediate oxygen species on CY and NT catalysts, respectively. The vertical solid lines represent the theoretical vibration band positions of O_2_^−^* (cyan) and OOH* (dark blue) and their relative intensities. **g**, **h** Time-resolved in situ ATR spectra over CY and NT, respectively. The data were shown as dots and the fitted lines were also presented. The peak positions were determined by the maximum intensity of O_2_ (ads) and O_2_^−^* bands. Reprinted with permission from Ref. [88]. Copyright 2022 Elsevier. (Color figure online)

More recently, different PAH nanocarbons comprising precise edge configurations and gradient structures to disclose the natures of carbon edges including establishment of structure–function relationships and observation of reactive intermediate during the H_2_O_2_ formation were reported by Lin and his colleagues(Fig. 10a) [88]. It can be found that the linear relationships among longitudinal size, number of outer carbon atoms, geometric area, and catalytic activity are plotted (Fig. 10b–d). To the best of our knowledge, it is the first time to establish a 2D structure–function relationship at a molecular level. With in situ ATR-IR spectra, DFT calculations and isotopic labeling, involved intermediate O_2_^−^* (cyan) located at 1163 cm^−1^ on CY (having armchair moiety) is identified and the common OOH* species are not observed evidenced by their non-reversible dynamic behaviors (Fig. 10e), suggesting that the latter mainly experiences a faster desorption step during the reaction. Moreover, the authors found that the location of O_2_^−^* (at 1206 cm^−1^) on NT with zigzag configuration is different with that of CY, but they have same features of comparative experiments. The different peak positions of O_2_^−^* can be explained by the steric-hindrance effect. These results demonstrate that the armchair and zigzag edge sites involve the same adsorbed intermediate oxygen species. This explains why they exhibit the similar ORR performance and same reaction pathway. Furthermore, the kinetic behaviors of two intermediates during the H_2_O_2_ formation were studied. As shown in Fig. 10g, h, O_2_ (ads) and O_2_^−^* species have different kinetics supported by their different equilibrium times. The former displays much faster formation rate during the reaction. Depending on different edge configurations, O_2_ (ads) and O_2_^−^* species show a steep growth trend in the first 7.3–10 s and reach equilibrium until 10–13.3 s, respectively. On either catalyst (CY or NT), the intensity of O_2_ (ads) is significantly higher than that of O_2_^−^* species. These kinetic behavior results reflect that the formation of O_2_^−^* species is closely associated with O_2_ (ads), which possibly determines the reaction selectivity. With the assistance of KIE, O_2_ (ads) + e^−^ → O_2_^−^* as a possible RDS is evidenced.

### OER

**Fig. 11:**
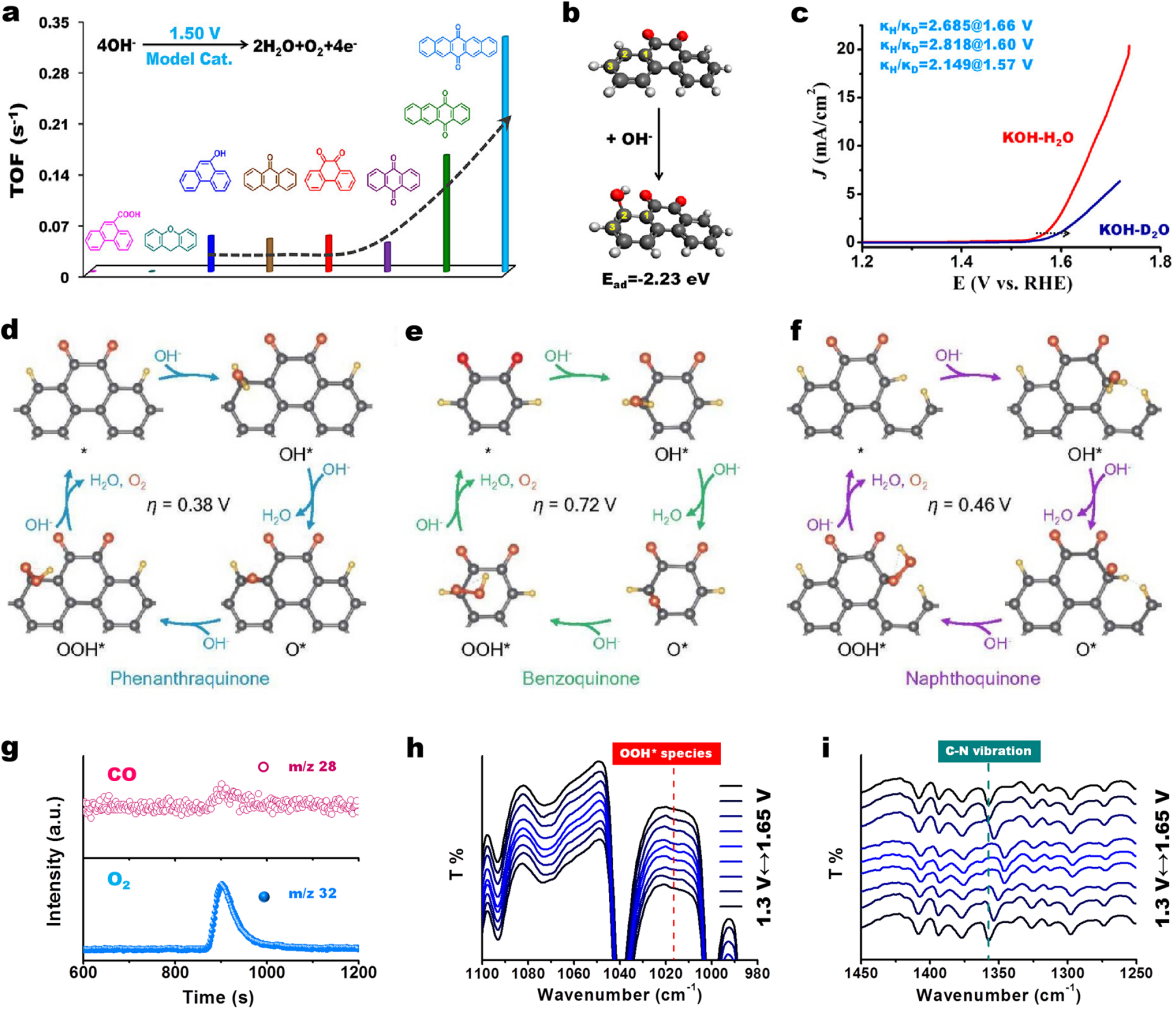
**a** Performance assessment of various model molecules with different O species (including quinone, hydroxy, ether, and carboxyl groups), edge configurations, and flexibly extended conjugated structure. **b** Adsorption energy and structure of OH^−^ species on model molecule having C=O group. Color code: carbon is gray, hydrogen is white, and oxygen is red. **c** KIE values calculated by current density ratio 0.1 M KOH dissolved in H_2_O and D_2_O (99.9%) solution. Reprinted with permission from Ref. [89]. Copyright 2018 American Chemical Society. **d–f** Consistency of theoretical and experimental activity and stability on phenanthraquinone, benzoquinone, and naphthoquinone, respectively. The gray, red, and yellow balls represent C, O, and H atoms, respectively. Reprinted with permission from Ref. [62]. Copyright 2018 American Chemical Society. **g** Mass spectra profiles of the produced CO (m/z 28) and O_2_ (m/z 32) with model molecules with isolated pyridinic N species. **h**–**i** In situ ATR-IR spectra for monitoring dynamic evolution of the involved intermediate *OOH and identifying the active sites on model molecules with isolated pyridinic N species. Reprinted with permission from Ref. [79]. Copyright 2021 Wiley–VCH. (Color figure online)

O is the most common alien atom for carbon materials as it is easy to adsorb at the edge sites of carbon network to generate different O species. The recent understanding for O species in OER is lack of reasonable evidence. Aromatic organic molecules with different O species as models were introduced to clarify the explicit roles of each common O group in OER at a molecular level [89]. The researchers found that edge (including zigzag and armchair) quinones in a conjugated *π* network are the true active centers, and the roles of ether and carboxyl groups are excluded in the OER process (Fig. 10a). Each C=O is demonstrated experimentally to contribute independently to the OER. C=O groups located at edge zigzag and armchair configurations show a similar contribution. With increasing the size of conjugated *π* network, the activity gradually improved, suggesting a directly proportional relationship between structure and function. An initial OH species is supposed to first adsorb at the meta-position (location 2) of the C=O group to achieve the first step of OER based on a negative adsorption energy (− 2.23 eV) of this process (Fig. 11b). With the assistance of isotopic labeling technique, the proton transfer concerted with the electron transfers in the proton-coupled electron transfer (PCET) system of model molecules was found (Fig. 11c). The deprotonation process of active oxygen species *OOH into OO^−^ species as a possible RDS in OER is singled out by H/D KIE values (Fig. 10d).

The similar results regarding the critical roles of C=O groups were also recently proposed by Jiao et al*.* [62]. With the calculations of the theoretical overpotentials at different possible model catalysts (Fig. 11d–f), it was found that phenanthraquinone, benzoquinone, and naphthoquinone show obviously smaller overpotentials than any other moiety. Phenanthraquinone moiety presents the lowest theoretical overpotential of only 0.38 V. Naphthoquinone takes second place at 0.46 V, and benzoquinone shows the largest value of 0.72 V, demonstrating the theoretical activity as phenanthraquinone > naphthoquinone > benzoquinone. Combination with the similar tendency behaviors of experimental TOF values, it is finally suggested that among the oxygen-containing functional groups, the ortho-quinone moieties are identified as the active sites.

More recently, the real contribution of each N species was revealed carefully toward OER also by using model molecules [76]. Pyridinic N species play a crucial role for improving the catalytic activity rather than carbon corrosion during the OER. This can be confirmed by using mass spectra, in which a very weak CO signal and an obvious O_2_ peak were observed (Fig. 11g). The location at edge zigzag or armchair positions of pyridinic N was proposed to be not a key to affect the catalytic performance. The dynamic behaviors of only OOH* species (without other species) were monitored experimentally, implying that the RDS mainly involves the evolution of OOH* species (Fig. 11h). Moreover, the red-shift of the C–N peak of pyridinic N at 1357 cm^−1^ with a potential-dependent tendency suggests that active sites should originate from adjacent carbon atoms (Fig. 11i). Based on these reported research work, we can therefore conclude that either molecule models or bulk models are very available for studying the activity origin and for detailing reaction pathways of carbon-based metal-free electrocatalysts for two-electron, four-electron ORR and OER.

### NRR

**Fig. 12:**
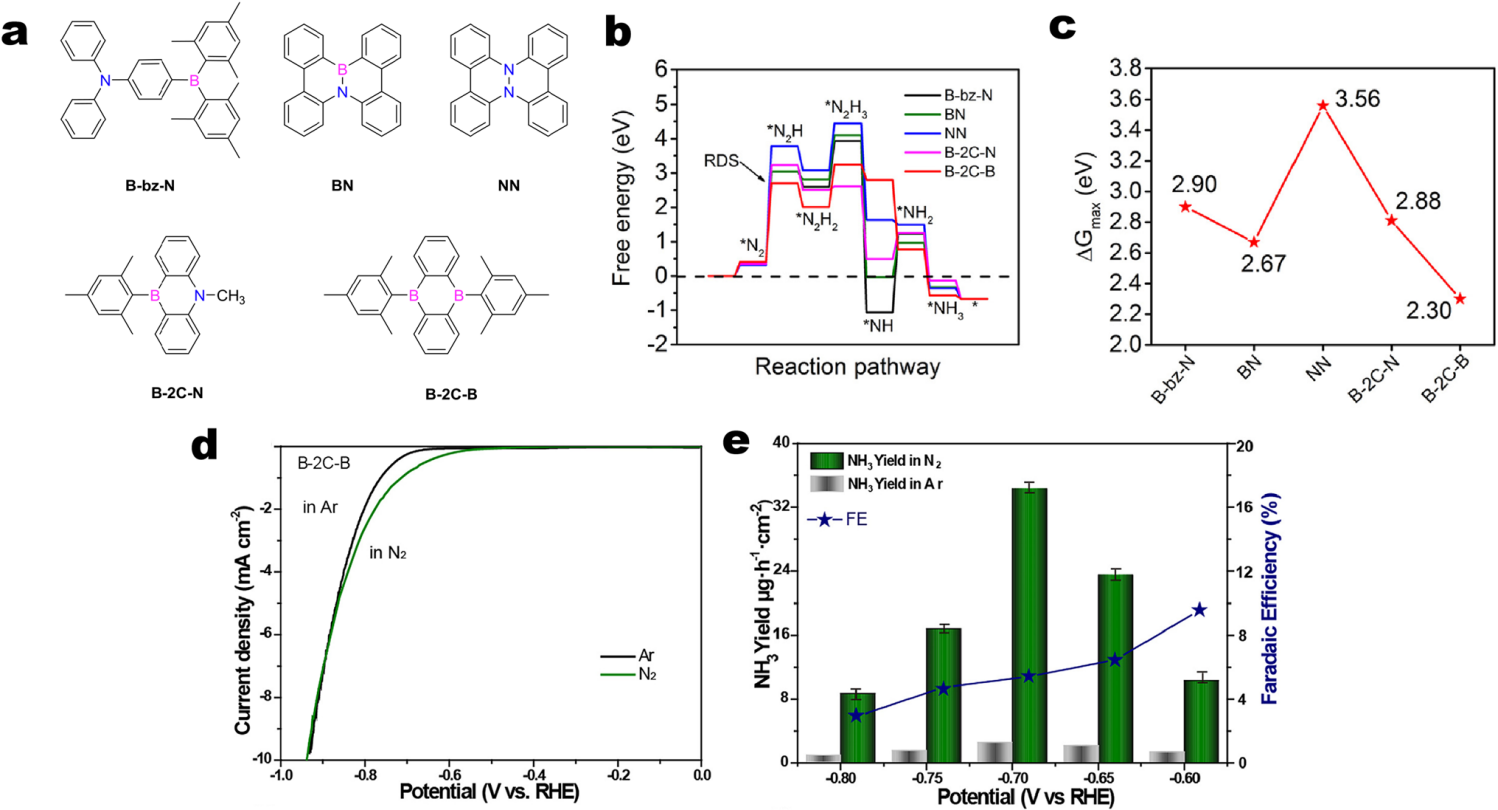
**a** Molecular structures of five polycyclic aromatic hydrocarbons: B–bz–N, BN, NN, B–2C–N and B–2C–B. **b** Full free energy diagrams of all these five PAHs. **c** The maximal Gibbs free energy changes. **d** LSV curves of B–2C–B in Ar (black curve) and N_2_ saturated electrolytes (green curve). **e** The NH_3_ production rates (left *y*-axis) and FE NH_3_ (right *y*-axis) of B–2C–B in Ar (grey column) or N_2_ saturating (green column) electrolytes at various applied potentials between − 0.6 and − 0.8 V. The error bars represent the average of three independent measurements. Reprinted with permission from Ref. [90]. Copyright 2020 Elsevier. (Color figure online)

Gu et al. developed several PAHs model catalysts with well-tuned number, position and separation of boron and nitrogen heteroatoms (Fig. 12a) [90]. The Gibbs free energy changes (Δ*G*) calculation showed that the first hydrogenation process to form intermediate of *N_2_H is the rate-determining step (RDS). B–2C–B, it exhibits the smallest value of the first hydro-genation free energy change (2.30 eV), suggesting that B–2C–B may serve as the most optimal electrocatalytic reduction of nitrogen reduction reactions (NRR) catalyst among these five PAH molecules (Fig. 12b, c). LSV test showed that At the potential window between − 0.65 and − 0.90 V, the B–2C–B catalyst presented a higher (more negative) current density in the N_2_-saturated electrolyte than in the Ar-saturated electrolyte (Fig. 12d). The optimally designed B–2C–B catalyst enabled a highest ammonia yield rate of 34.58 μg h^−1^ cm^−2^ and an excellent FE_NH3_ of 5.86% at an applied potential of − 0.7 V versus RHE (Fig. 12e). These results provide a new perspective on the involvement of metal-free carbon-based catalysts for N_2_ electrochemical fixation.

## Conclusion and Perspective

In conclusion, the present Review provides an overview of the recent progress of model catalysts in mechanistic studies and key intermediates on metal-free carbon-based thermo- and electro-catalytic reactions. The conceptual knowledge in these fields, such as the identity of the active sites, reaction mechanisms, and relatively precise structure–function relations, have been revealed with a promising model method coupled with in situ characterization techniques. Ketonic carbonyl groups are identified as the catalytic active sites on nanocarbon for EB ODH reactions by using model molecules, and the quantity of the active sites, the intrinsic catalytic activity (TOF), and kinetic behaviors of nanocarbon could be measured through in situ chemical titration method, in situ FT-IR measurements, and isotopic studies. Quinone species and zigzag edge configuration instead of common O species and armchair configuration is proposed to be responsible for cross-dehydrogenative coupling reactions. Different model molecules with exclusive N species were used to reveal the exact role of each common N species in alcohol oxidation. Pyridinic N species play an unexpected role in manipulating catalytic process. The activation process of the α-H of –CH_2_OH is clarified to be an RDS of the aerobic alcohol oxidation reaction using KIE. Neighboring carbon atoms in pyridinic N species are responsible for facilitating the RDS process. Moreover, with regard to electrocatalytic reactions involving controversial scientific issues, the real contributions and activation processes of N species are studied. Pyridinic N species dominate the alkaline and acidic ORR via a 4–e^−^ pathway. Pyridinic N groups located at zigzag and armchair edge configurations show similar catalytic performance, and each pyridinic N is demonstrated experimentally to contribute independently to the ORR reaction. The nearly linear structure–function relationship can be established by delicately controlling the longitudinal extension (*π*-conjugated structures), local environment, and distribution density. Key intermediates, including adsorbed O_2_ molecules, OOH* and O_2_* species, can be experimentally identified and monitored. O_2_^−^* + H_2_O → OOH* + OH^−^ as a possible RDS during the ORR process is proposed. The hydrophobic environment promoted by pyridinic N is associated with enhanced ORR activity. It should be noted that O_2_ (ads) + e^−^ → O_2_^−^* is evidenced to a possible RDS for H_2_O_2_ electrosynthesis. In the case of two kinds of heteroatoms doping, B, N dopants with no direct bonding are beneficial to the generation of two electrophilic active sites in close proximity for bidentate O_2_ binding. Furthermore, with the help of model molecules with singly different O and N species, both C=O group and pyridinic N species are suggested to be positive in accelerating the OER. The generation of O_2_ from OOH* species is proposed to be the most likely RDS during the OER process. The carbon catalysts co-doped with boron and nitrogen for NRR, B tends to be the active center compared to C.

There are still the following shortcomings in the current model catalyst research: (i) the scale of the conjugated system of the model catalyst studied so far is still small; (ii) the synthesis of large PAHs containing between 30 and 100 conjugated carbon atoms will be challenging due to their low solubility; (iii) the heteroatom substitution of the specific carbon atom of PAHs requires a new synthesis route, which has certain difficulties and affects its application in catalysis. More efficient synthesis methods of model catalysts need to be developed. Therefore, there is still a lot of work to be done before this fascinating class of nanocarbon molecules can be brought to light for real practical applications. The following aspects we believe are the possible research directions of model catalysts for metal-free carbon catalysis.

(i) Gradient nanocarbon molecule catalysis. As mentioned above, a 2D structure–function relationship can be established by delicately controlling longitudinal extension (*π*-conjugated structures) of model molecules. The used model molecules themselves also show promising performance toward some typical reactions. Based on that, we propose a conceptual outlook regarding a ‘gradient nanocarbon molecule catalysis’ branch by concentrating on the precise structure and size designs of model molecules with high activity. Depending on catalytic reaction types, model molecules with tunable acid–base properties are designed via the editing of *π*-conjugated structures and the incorporation of alien species. It can be expected that such gradient molecules with designedly single active component would become an emerging and conceptually new area of metal-free carbon catalytic materials. A series of gradient relationship networks between structure and function can be built. A new family of nanocarbon molecule catalysts beyond common carbon materials will be found.
(ii) Time-resolved observation of dynamic behaviors of key intermediates at a single molecular level, even at an atomic level. Although model catalysts provide an important platform to study catalytic mechanism at a molecular level, it is still difficult to observe dynamic evolution of transient key intermediates at a higher resolution level (e.g., microsecond class). Developing the time-resolved operando technique at a single molecular level (e.g., PAH nanomolecules consisting of above 100 carbon atoms), even at an atomic level (e.g., PAH nanomolecules doped with heteroatoms), is of importance to deeply understand the detailed reaction kinetics at the interfaces between catalysts and reactive media, the transient adsorption and desorption behaviors of reactants and products and the mass transfer pathway. It could offer key reference information for the design of carbon-based catalysts and for insight into catalytic nature.
(iii) Experimental uncovering of reaction pathways. Aromatic organic molecules are good model catalysts to reveal reaction pathways as they have the well-designed structure, the homogeneous active sites, and the known electron donor–acceptor properties. These advantages are favorable to differentiating electron transfer (ET), proton transfer (PT) and PCET for typical electrocatalytic reactions in combination with isotopic studies. Taking OER and ORR as examples, given that the recently common RDS revealed by DFT calculations (based on PCET mechanism), the relevant experiments should be carried out to prove the real elementary reaction step, the real contribution of each ET, PT, and PCET process and the most likely RDS.
(iv) Study on the structural evolution of metal-free carbon catalysts during electrocatalysis. Recent research has found that N heteroatoms can be oxidized by high potentials during OER, producing oxoanions. In the HER process, it is hydrogenated and reduced to ammonia. To further elucidate the specific mechanism of this process, model molecules can be designed to evaluate this process. Through this method, it is expected to analyze the impact of this process on the structure and performance of the catalyst at the atomic level.
(v) The model carbon can not only be used as a metal-free catalyst, but also be used as promising supports to anchor the metal atom to boost the catalytic activity. The interaction between precise structure of nanocarbon and metal atoms, as well as the synergistic catalytic effect, are worthy of further in-depth study.

This Review forecasts a promising future in metal-free carbon catalysis by model materials. We believe that the applications of this special kind of carbon molecules can be extended to organic synthesis and energy chemistry and thus have an unique perspective in chemistry.
